# Animal-based folk remedies sold in public markets in Crato and Juazeiro do Norte, Ceará, Brazil

**DOI:** 10.1186/1472-6882-9-17

**Published:** 2009-06-03

**Authors:** Felipe S Ferreira, amuel V Brito, Samuel C Ribeiro, Antônio AF Saraiva, Waltécio O Almeida, Rômulo RN Alves

**Affiliations:** 1 Universidade Regional do Cariri, Departamento de Química Biológica, Crato, CE, Brazil; 2 Universidade Federal do Pernambuco, Departamento de Zoologia, Recife, PE, Brazil; 3 Universidade Regional do Cariri, Departamento de Ciências Físicas e Biológicas, Crato, CE, Brazil; 4 Universidade Estadual da Paraíba, Departamento de Biologia, 58109-753, Campina Grande, PB, Brazil

## Abstract

**Background:**

Human communities consistently develop a detailed knowledge of the therapeutical and medicinal properties of the local flora and fauna, and these folk remedies often substitute medicines produced by the pharmaceutical industry. Animals (and their derived products) are essential ingredients in the preparation of many traditional remedies. The present work prepared an inventory of the animals sold in public markets in the cities of Crato and Juazeiro do Norte, Ceará State, Brazil.

**Methods:**

Information was obtained through the use of semi-structured questionnaires in interviews held with 27 merchants of medicinal animals (18 in the municipality of Juazeiro do Norte [11 men and 7 women] and 9 people in the municipality of Crato [6 men and 3 women]). We calculated the Informant Consensus Factor (ICF) to determine the consensus over which species are effective for particular ailments, as well as the species Use Value (UV) to determine the extent of utilization of each species.

**Results:**

A total of 31 animal species, distributed among 21 families were identified as being used medicinally. The taxa most represented were: insects (8 species), mammals (7), fish (5), reptiles (5) and birds (4). The animals sold in these markets are used to treat a total of 24 ailments, with rheumatism, asthma, and inflammations having the largest numbers of citations. Three species not previously reported as having medicinal use were encountered: *Leporinus steindachneri *(utilized for treating cholesterol problems), *Gryllus assimilis *(utilized in treating urinary infections), and *Phrynops tuberosus *(used to treat asthma, rheumatism and bruises).

**Conclusion:**

The composition of the local fauna, the popular culture, and commercial considerations are factors that maintain and drive the market for therapeutic animal products – and the lack of monitoring and regulation of this commerce is worrisome from a conservationist perspective. A detailed knowledge of the fauna utilized in alternative medicine is fundamental to the conservation and rational use of the Brazilian fauna.

## Background

The use of medicinal plants and animals in folk medicines are ancient practices, and they are based on popular knowledge transmitted from generation to generation. Human communities have developed a wide practical knowledge of the therapeutical and medicinal properties of many animals and plants, and the use of these natural resources as folk remedies often substitute for medicines produced by the pharmaceutical industry with prices well above their economic reach [1].

Animals (and their derived products) constitute essential ingredients for the preparation of traditional remedies that have been used since pre-historic times [2-5]. The use of animals in popular medicine is a widely distributed phenomenon from both a historical and geographical view point, and this use has been studied by workers in many different fields – ethnography [6,7] medicine [5,8], pharmacology [9], and ecology [5,10-15]. Many different animal species have been reported as being used for medicinal purposes since the first European colonists arrived in Brazil, and they offer many therapeutical options [5,16-19]. A recent review of the subject reported 290 different animal species being used in traditional folk medicine in Brazil – although this number may turn out to be much higher as more research is undertaken [20]. The scarcity of research focusing on zootherapy has contributed to an underestimation of the importance of zootherapeutic resources in Brazil, although studies in this area have become more prevalent in recent years, at least at a global level.

The use of medicinal animals can be observed in both rural and urban settings in Brazil, being sold by "*erveiros*" (herb merchants) in public markets throughout the country [8,21-24]. It is common to find specific locations in these markets where plants and animals are sold for medicinal purposes, locations that serve to unite, maintain, and diffuse empirical knowledge from different regions and of different origins [17,25]. Almeida and Albuquerque [23] have proposed that information obtained from people working in these commercial centers can be used to formulate rational strategies for the commercialization and sustainable use of these important natural resources.

In light of the fact that many of the animal species sold for medicinal use are listed as rare or threatened species [5], there are ecological as well as cultural, social, and public health implications associated with their use, and more complete inventories of these animals are needed and closer examinations of the socio-cultural contexts of their use.

The use of zootherapeutics is a very common practice in northeastern Brazil [21,26]. The traditional knowledge associated with the folk use of animals has been transmitted through many generations and now has an important role in public health by offering accessible alternative treatments for common aliments. Due to the limited access of poor people to health services (and their low quality), zootherapeutic medicines may offer inexpensive and easily obtainable alternatives to commercial pharmaceutical remedies [5].

Most research on zootherapy in northeastern Brazil has been undertaken in rural areas although there are still a number of states in which this theme has never been investigated [21]. Ceará state, where the cities of Juazeiro do Norte and Crato are located, has never hosted a zootherapeutic study – even though the use of traditional medicine is very common in the region. The present work investigated the commercialization of medicinal animals in public markets in the cities of Crato and Juazeiro do Norte in order to examine the following questions: i) what medicinal animal species are being sold? ii) What animal parts are used? iii) What ailments are treated with animal-based medicines?

## Methods

### Study Area

**Figure 1:**
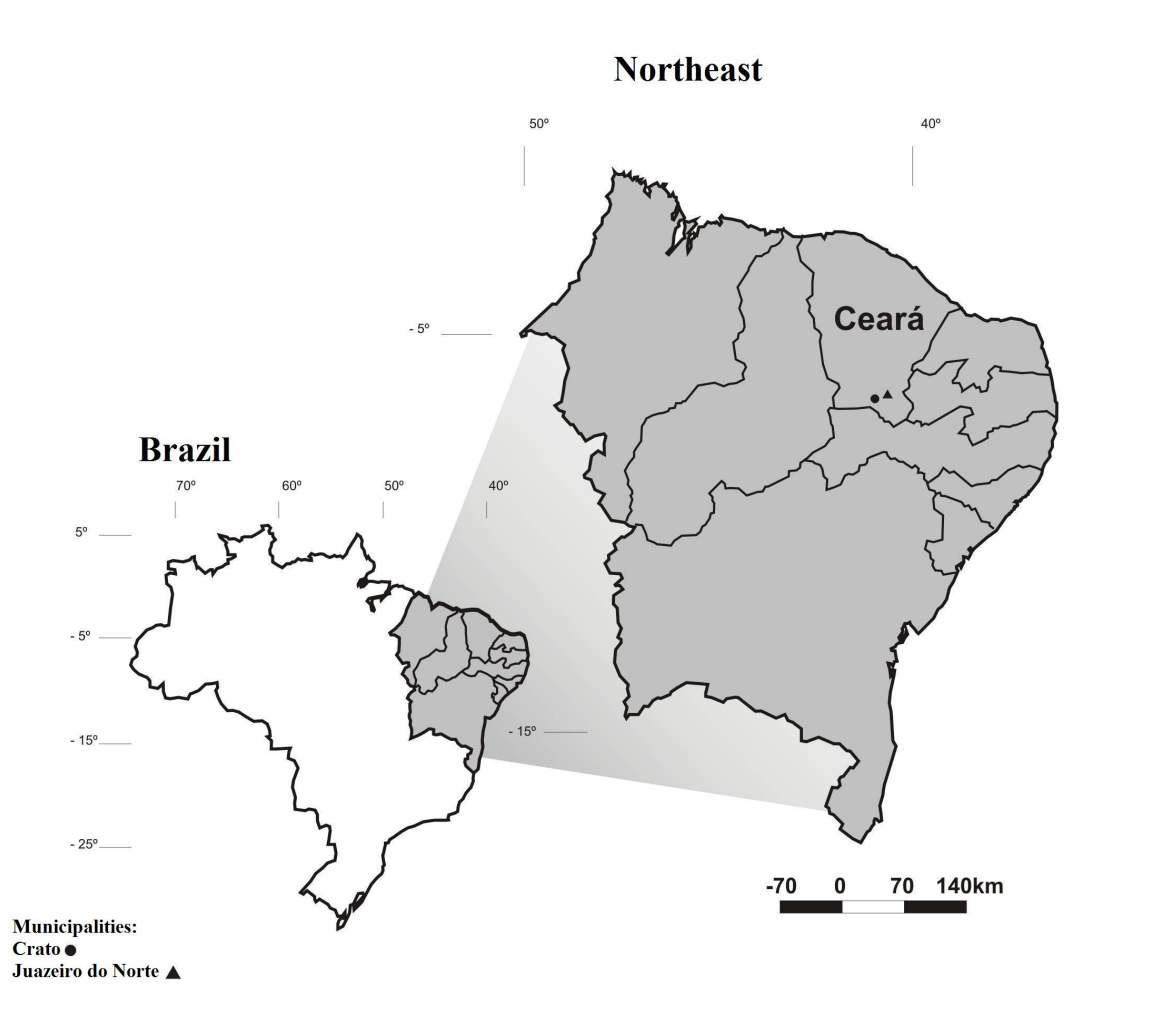
**Map locating the cities studied in Ceará State, Brazil**.

The municipalities of Crato (7° 14' 03" S × 39° 24' 34" W) and Juazeiro do Norte (7° 12' 47" S × 39° 18' 55" W) are located in the southern part of Ceará State, Brazil (Figure 1). The municipality of Crato occupies an area of approximately 1,009 Km^2 ^and has approximately 111,000 inhabitants [27]. The municipality de Juazeiro do Norte occupies an area of approximately 249 Km^2 ^and has approximately 242,000 inhabitants [27]. The principal economic activities of Crato are related to commerce and tourism, and the municipality has six hospitals and 20 health centers [28]. The principal economic activities of Juazeiro do Norte are related to commerce and religious tourism, and the municipality has four hospitals and 50 health centers [28].

The two municipalities are located in the center of the Northeast, in contact with the borders of neighboring states, showing different aspect in the socio-cultural context of a culture of Ceará resulted of influences from socio-economic approaches and bordering states of Pernambuco, Paraíba and Piauí [29]. Juazeiro do Norte and Crato have an important socio-cultural relevance observed in tourism due to religious pilgrimages, held since 1934, in honor of Padre Cicero [29,30].

These two municipalities are located in the Araripe bio-region, which is composed of a mosaic of ecosystems including humid forests (Sub-perennial Tropical Pluvial-Nebular Forest), dry forests (Sub-deciduous Tropical Pluvial Forest), *cerradão *(Sub-deciduous Tropical Xeromorphic Forest), *caatinga *(Deciduous Thorn Forest), *carrasco*, and *cerradão *[28,31].

The faunal composition of the Araripe bio-region includes common vertebrate taxa such as mammals [32], birds [33], and reptiles [34]; while the most well investigated invertebrate taxa include nematodes [35] and pentastomids [36].

### Procedures

Field work was undertaken during the period from June to August, 2008, in public markets in Crato and Juazeiro do Norte where products derived from animals and plants are widely sold for therapeutic purposes. Twenty seven shop owners selling animal and plant-based medicines were interviewed (18 in the municipality of Juazeiro do Norte [11 men and 7 women] and 9 people in the municipality of Crato [6 men and 3 women]). This was a pre-defined and intentionally non-random sampling of interviewees [37] composed only of people who actually sold zootherapeutic products.

To respect intellectual property rights, we adopted the following protocol in the field: before the survey, we introduced ourselves, explained the nature and objectives of our research and asked the respondents for permission to record the information. The ethical approval for the study was obtained from the Ethics committee of Faculdade de Medicina de Juazeiro do Norte – FMJ (N° of protocol: 2009-0319CEP).

Semi-structured questionnaires were filled out during the unstructured interviews and informal conversations, and they contained questions about the medicinal use of animal species, their respective uses, preparation, and the body parts utilized.

Vernacular names of species were recorded as quoted during the interviews. Zoological material was identified with the aid of specialists, through examination of voucher specimens donated by the interviewees or purchased at the surveyed markets, and through photographs taken during interviews of the animal species or their parts. Whenever necessary, these procedures were supplemented by checking vernacular names provided by traders against the scientific names, with the aid of taxonomists familiar with the study areas. All specimens that were collected, as well as photographs of the specimens described, were deposited in the Laboratório de Zoologia of Universidade Regional do Cariri – URCA.

### Data Analysis

The ailments treated by zootherapeutics were grouped into 11 categories (Table 1) based on the model used by the "Centro Brasileiro de Classificação de Doenças" (Brazilian Center for the Classification of Diseases) [38].

**Table 1:** Categories of diseases treated with animal-based medicines that are sold in public markets in Crato and Juazeiro do Norte, according to the "Centro Brasileiro de Classificação de Doenças" (1993)

Categories	Ailments cited by the vendors	Total
A	Inflammations and "simpatias"	3
B	Asthma, coughs, sore throat and bronchitis	4
C	Rheumatism, arthritis, back aches and osteoporosis	4
D	Hemorrhoids and cholesterol	2
E	Snake bites	1
F	Ear aches and deafness	2
G	Urinary infections	1
H	Bruises and alcoholism	2
I	Intestinal infections	1
J	Whooping cough, pityriasis and leprosy	3
K	Fissures on the sole of the feet	1

Total	24	

A: undefined illnesses; B: respiratory system; C: osteomuscular system and conjunctive tissue; D: circulatory system; E: Lesions caused by poisoning and other external causes; F: diseases of the ear; G: diseases of the urogenital system; H: diseases due to external causes; I: digestive system; J: Infections or diseases caused by parasites; K: skin and the subcutaneous tissue.

To estimate the level of agreement between interviewees over which animals to use for each category, we calculated the informant consensus factor (ICF), adapted from Heinrich et al. [39] that looks at the variability of animals used for each treatment, and therefore the consensus between practitioners. This factor estimates the relationship between the "number of use-reports in each category (*n*ur) minus the number of taxa used (*n*t)" and the "number of use-reports in each category minus 1". ICF is thus calculated using the following formula:



The product of this factor ranges from 0 to 1. A high value (close to 1) indicates a high consensus whereby relatively few taxa (usually species) are used by a large proportion of people, while a low value indicates that the informants disagree on the taxa to be used for treating a particular illness.

### Use-value

For each species the use-value (UV), adapted from the proposal of Phillips et al., [40], was calculated. This quantitative method evaluates the relative importance of each medicinal species based on its relative use among informants. These values were calculated using the following formula: UV=Σ*U*/*n*, where U is the number of citations per species and *n *is the number of informants. The use-value of each species is therefore based objectively on the importance attributed by the informants and does not depend on the opinion of the researcher.

## Results and Discussion

The commercialization of zootherapeutic medicines was observed to be a common practice in Crato and Juazeiro do Norte. A total of 31 species, distributed among 21 families, were found being sold for medicinal purposes in these cities (Additional file 1). The taxa most commonly sold were insects (8 species), mammals (7), fish (5), reptiles (5), and birds (4). These results represent the first data available concerning the use and commercialization of animals in folk medicine in Ceará State, and they corroborate with the results of other studies that have found the sale of zootherapeutic items to be a common activity in the semi-arid region of northeastern Brazil [20,21,23,41].

The total of 31 medicinal species sold in Crato and Juazeiro do Norte is quite expressive in comparison with results presented by other workers who examined public markets in northeastern Brazil. A total of 17 animal species used for medicinal purposes were identified in markets in the city of Maceió [42], 16 species in Feira de Santana [22], 18 species in Caruaru [23], 37 species in Santa Cruz do Capibaribe [41], and 18 species in Recife [24]. Most of the species sold in Crato and Juazeiro do Norte were same as in other towns in the northeast, but three new species were identified: *Leporinus steindachneri*, *Gryllus assimilis *and *Phrynops tuberosus*. The body fat from *L*. *steindachneri *is used to treat problems with cholesterol, the legs of *G*.*assimilis *are used to treat urinary infections, and the shell and body fat of *P. tuberosus *are used to treat asthma, rheumatism and bruises respectively.

**Figure 2:**
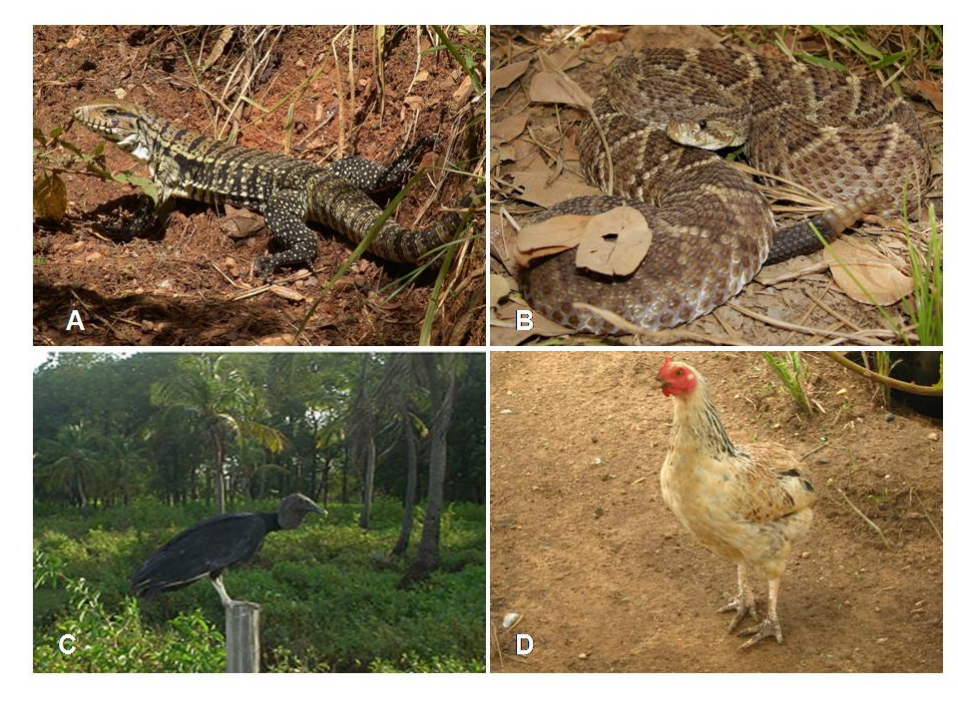
**Examples of animals used as medicine that are sold in public markets in Crato and Juazeiro do Norte**. A: *Tupinambis merianae*, B: *Crotalus durissus *(Photo: Daniel Loebmann), Gallus domesticus (Photo: Samuel C Ribeiro), D: *Coragyps atratus *(Photo: Romulo RN Alves).

The species most frequently cited in the present study were: *Tupinambis merianae *(n = 20) and *P. tuberosus *and *Crotalus durissus *(n = 18) (Figure 2). These species are similarly widely used in alternative medicinal practices in other regions of Brazil [16-18,22,41]. Most of the animal species cited are only found wild in nature; only five domesticated animals were found to be used for medicinal purposes: *Gallus domesticus*, *Pavo cristatus*, *Ovies aries*, *Bos taurus *and *Sus scrofa*.

All of the animals found being sold were native to the Araripe bio-region, with the exception of *Oreaster reticulatus*, *Hippocampus reidi *and *Caiman crocodilus*. This finding demonstrates the importance of local biodiversity in furnishing folk medicines, in agreement with Alves & Rosa [16] who observed that faunal composition, accessibility, and availability directly influence the types of zootherapeutic items sold in any given region. The use of the local fauna generally reduces the acquisition costs of commercial agents, and our results are in agreement with Apaza et al. [43], who noted a reduction in the cost of acquiring animal products in regions with abundant faunal resources.

The commercialization of a number of species not native to the semi-arid region of northeastern Brazil was also noted in other studies, indicating the existence of extensive commercial routes for animals harvested in other biomes [18,44]. Some of the species sold in Juazeiro and Crato are listed as threatened with extinction. *H. reidi*, for example, is a threatened species commonly used for treating asthma.

**Figure 3:**
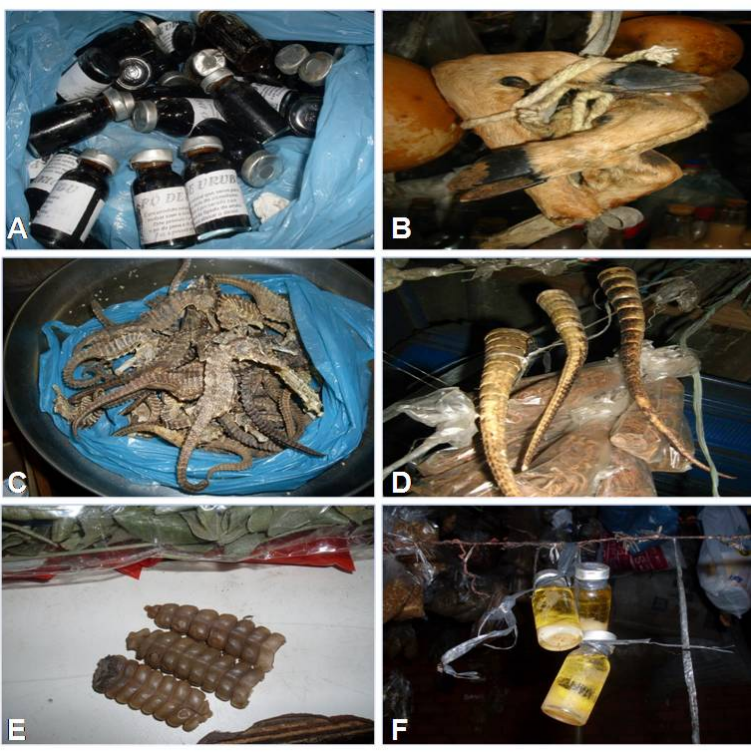
**Examples of animal products used as remedies in that are sold in public markets in Crato and Juazeiro do Norte**. A: Liver powder of *Coragyps atratus*, B: Hoof of *Mazama *sp., C: Dried Seahorses (*Hippocampus reidi*), D: Tail of *Euphractus sexcinctus*, E: Rattle of *Crotalus durissus*, F: Fat of *Tupinambis merianae *(Photos: Samuel C Ribeiro).

Various animal parts and products are used in the preparation of folk medicines, including: honey, wax, urine, horns, viscera (stomach and liver), fat, marrow, skin, feathers, legs, tails, navels, and hooves (Figure 3). Small animals such as *Dinoponera quadriceps*, *Tropidurus hispidus*, *Crotophaga ani, O*. *reticulatus *and *H*. *reidi *may be used whole. Two of the most widely used products are honey produced by *Apis mellifera *and the body fat of *T*. *merianae*.

Animals provide the raw materials for remedies prescribed using the clinical method and are also used in the form of amulets and charms in magic-religious diagnosis. The use of some medicinal animals is associated with popular beliefs locally known as '*simpatias*'. These '*simpatias*' are often secretive in nature, so that the people receiving the treatment cannot know what that they are taking, otherwise the remedy will not be effective. This popular belief is commonly associated with the use of medicinal animals in Brazil [5].

Some species have multiple uses, and are utilized for treating more than one type of ailment. The fat from *C. durissus*, for example, is used to treat rheumatism, osteoporosis, leprosy, back-aches, ear-aches, and fissures on soles of the feet; while the rattle from this same snake is used in *simpatias*. The fat of *T. merianae *is used to treat rheumatism, inflammations, fissures on sole of the feet, and ear-aches. The shell of *P. tuberosus *is used to treat asthma, while its fat is used in to treat rheumatism and bruises. The fat from *Hoplias malabaricus *is utilized in the treatment of inflammations, urinary infections, and ear-aches.

The zootherapeutic item cited with the greatest frequency was animal fat, a product that can be extracted from the following animals: *H. malabaricus*, *L. steindacheneri*, *Eletrophorus electricus*, *T. merianae*, *P*.*tuberosus*, *C. durissus*, *G. domesticus*, *Cerodcyon thous *and *O. aries *– all of which are vertebrates and can produce and store large amounts of this substance [41].

The interviewees in the Crato and Juazeiro do Norte markets indicated that zootherapeutic products are prescribed for at least 24 different illnesses. The categories with the greatest ICF values were: osteomuscular system and conjunctive tissue (0.9), undefined diseases (0.81), ailments caused by external agents (0.77), and respiratory system (0.76). The high ICF values for these categories corroborate results from other public markets in northern and northeastern Brazil [17]. The species with the highest use-values in the categories osteomuscular system and conjunctive tissue, undefined diseases, and respiratory system was *T. merianae *(UV = 0.74), while in the category of ailments caused by external agents, the species with the highest use-value was *P. tuberosus *(UV = 0.66). The high number of use citations in category osteomuscular system possibly result from the fact that the fats obtained from 9 of the species quoted are most the usual medicinal resources, and are recommended to treat a variety of diseases, mainly the diseases belonging to osteomuscular system (e.g., rheumatism and backache).

A total of 132 citations of uses for medicinal animal products were cataloged (Table 2). The categories with the largest numbers of citations were: respiratory system (64 citations; 16 species), osteomuscular system and conjunctive tissue (51 citations; 6 species), and undefined diseases (34 citations; 7 species). A few categories had very few citations, such as: circulatory system (4 citations; 3 species), diseases and infections related to parasites (4 citations; 3 species), skin and of subcutaneous tissues (4 citations; 2 species), and lesions and other external consequences caused by poisoning (1 citation; 1 species).

**Table 2:** Consensus factors of the informants for the categories described

	Categories										
	
	A	B	C	D	E	F	G	H	I	J	K
Juazeiro do Norte											
Species used	7	11	5	3	1	7	1	2	3	1	2
Percentage of species used (%)	30.4	47.8	21.7	13.0	4.3	30.4	4.3	8.6	13.0	4.3	8.6
Use citations	25	45	39	3	1	20	1	8	3	1	4
Percentage of use citations (%)	29.0	52.3	45.3	3.4	1.1	23.2	1.1	9.3	3.4	1.1	4.6
ICF	0.79	0.77	0.89	0	0	0.68	0	0.85	0	0	0.66
Crato											
Species used	5	12	5	1	-	3	3	1	3	2	-
Percentage of species used (%)	20.8	50	20.8	4.1	-	12.5	12.5	4.1	12.5	8.3	-
Use citations	9	19	12	1	-	3	5	2	3	3	-
Percentage of use citations (%)	19.5	41.3	26.0	2.1	-	6.5	10.8	4.3	6.5	6.5	-
ICF	0.5	0.38	0.63	0	-	0	0.5	1	0	0.5	-
All localities combined											
Species used	7	16	6	3	1	7	3	3	3	3	2
Pecentage of species used (%)	22.5	51.6	19.3	9.6	3.2	22.5	9.6	9.6	9.6	9.6	6.4
Use citations	34	64	51	4	1	23	6	10	6	4	4
Percentage of use citations (%)	25.7	48.4	38.6	3.0	0.7	17.4	4.5	7.5	4.5	3.0	3.0
ICF	0.81	0.76	0.9	0.33	0	0.68	0.6	0.77	0.6	0.33	0.66

The diseases with the greatest numbers of citations by merchants working in public markets in Crato and Juazeiro do Norte were: rheumatism (40 citations; 30.3%), asthma (27 citations; 20.4%) and inflammations (26 citations; 19.6%). Earlier research performed in the northeastern region of the country likewise indicated these same diseases as being treated with animal-based medicines [1,17,18,23,44].

Folk remedies are usually prepared in the follow manners: whole animals or body parts are macerated and the resulting powder is consumed in the form of teas or it is ingested together with food; secretions (urine) and fats are administered as ointments or are ingested. All interviewees prescribe the same forms of preparation zootherapeutic. There were no reports of zootherapeutic substances being used together with other alternative medicines.

Many animals sold in the markets in Crato and Juazeiro do Norte were used to treat "spiritual" ailments; *Oreaster reticulatus *(a star fish), the rattle of the rattlesnake *Crotalus durissus*, and the horn of *Bos taurus *or the hooves and tails of *Mazama americana *are used to ward off the "evil-eye". The use of some species are associated with popular beliefs ("simpatias"), for example, the tails of *Euphractus sexcinctus *or *Dasypus novemcinctus *are introduced into the ear to treat deafness. These treatments are much the same as those reported from the city of Santa Cruz do Capibaribe in Pernambuco State [41]. This information corroborates research by Alves et. al [5] who pointed out that the use of animal-based medicines is closely associated with local beliefs in many different regions of Brazil. In addition to their medicinal uses, some zootherapeutic products (such as the rattle of *C*.*durissus*) are also used in the Afro-Brazilian religious rituals of "Candomblé".

According to the interviewees, animal-based medicines are more effective and less expensive than industrially produced pharmaceuticals. Zootherapy is very important and wide-spread in Brazil, and more studies will be needed to examine the true efficiency of these remedies. Additionally, it is important to stress that a significant percentage of the species commercialized for medicinal purposes are wild animals, and many of them must be sacrificed in order to obtain the desired zootherapeutic products. High-demand species with high commercial value are most susceptible to over-exploitation, and multidisciplinary studies will be needed to examine the social, cultural, economic, clinical, and environmental aspects of their consumption and to establish adequate management strategies that can help guarantee the sustainable use of these zootherapeutic resources.

## Conclusion

The present work recorded the use and sale of 31 species of animals used as folk medicines in the cities of Crato and Juazeiro do Norte. These animals or their products are used to treat 24 different ailments – with rheumatism, asthma, and inflammations receiving the largest numbers of citations. The composition of the local fauna, the popular culture, and commercial considerations are factors that maintain and drive the market for therapeutic animal products – and the lack of monitoring and regulation of this commerce is worrisome from a conservationist perspective.

A well-founded knowledge of the fauna utilized in folk medicine is a requisite for the conservation and rational use of these natural resources. Only intensive field research will be able to determine practical approaches to the management and sustainable use of the local fauna for utilitarian purposes, and local traditional knowledge will have a very important role in this process – for economic development and the rational use of biodiversity are inextricably linked.

## Competing interests

The authors declare that they have no competing interests.

## Authors' contributions

FSF, SVB, RRNA and WOA – Writing of the manuscript, literature survey and interpretation; FSF, AAFS and SCR-Ethnozoological data, literature survey and interpretation; SCR and AAFS – Analysis of taxonomic aspects. All authors read and approved the final manuscript.

## Pre-publication history

The pre-publication history for this paper can be accessed here:



## Supplementary Material

Additional file 1 **Animal species commercialized for medicinal purposes in the municipalities of Crato and Juazeiro do Norte, Ceará, Brazil**. The data provided animal species commercialized for medicinal purposes in the municipalities of Crato and Juazeiro do Norte, Ceará, Brazil. Click here for file
