# Supplementary material for: Animal-based folk remedies sold in public markets in Crato and Juazeiro do Norte, Ceará, Brazil
Source: BMC Complement Altern Med. 2009 Jun 3;9:17. doi: 10.1186/1472-6882-9-17 (PMC2698880; doi:10.1186/1472-6882-9-17)
Supplement: Additional file 1 — Animal species commercialized for medicinal purposes in the municipalities of Crato and Juazeiro do Norte, Ceará, Brazil. The data provided animal species commercialized for medicinal purposes in the municipalities of Crato and Juazeiro do Norte, Ceará, Brazil. [file 1472-6882-9-17-S1.doc]

**Additional files
Additional file 1**

**File format: DOC
Title - Animal species commercialized for medicinal purposes in the municipalities of Crato and Juazeiro do Norte, Ceará, Brazil**

**Description: The data provided animal species commercialized for medicinal purposes in the municipalities of Crato and Juazeiro do Norte, Ceará, Brazil**

| Family/species/ local name | Number of citations | | Use-value | | Part used | Disease (or illness) |
| --- | --- | --- | --- | --- | --- | --- |
|  | JN | CR | JN | CR |  |  |
| **Insects**  Formicidae  *Dinoponera quadriceps* (Santschi,1921) - ant, “formigão” | 4 | - | 0.22 | - | Whole animal (1) | Ear aches |
| Blattidae  *Periplaneta americana* (Linnaeus, 1758) - cockroach, “barata” | 1 | 1 | 0.05 | 0.11 | Viscera, wings (2) | Ear aches (2) and asthma (2) |
| Gryllidae  *Gryllus assimilis* Fabricius, 1775 - cricket, “grilo” | 1 | 2 | 0.05 | 0.22 | Leg (2) | Urinary infections |
| Apidae  *Melipona scutellaris* (Latreille, 1811) - stingless bee, “uruçú” | 1 | 1 | 0.05 | 0.11 | Honey (3) | Coughs |
| *Partamona cupira* (Smith) - stingless bee, “cupira” | 1 | 1 | 0.05 | 0.11 | Honey (3) and wax (4) | Coughs (3) and stomach ache (4) |
| *Apis mellifera*(Linnaeus, 1758) - honey bee, abelha italiana” | 1 | 2 | 0.05 | 0.22 | Honey (3) and wax (4) | Coughs (3) and stomach ache (4) |
| *Melipona subnitida* (Ducke, 1910) - stingless bee, “jandaíra” | 1 | 1 | 0.05 | 0.11 | Honey (3) and wax (4) | Coughs (3) and stomach ache (4) |
| Curculionidae  beetle, besouro bicudo | - | 2 | - | 0.22 | Larvae (2) | Asthma and coughs |
| **Equinodermes**  Oreasteridae  *Oreaster reticulatus* (Linnaeus 1758) **-** starfish, “estrela do mar” | 2 | - | 0.11 | - | Whole animal (1,6) | Asthma (1) and “simpatias” (6) |
| **Fish**  Erythrinidae  *Hoplias malabaricus* (Bloch, 1794) - trahira, “traíra” | - | 3 | - | 0.33 | Fat (5) | Inflammations, urinary infections and ear aches |
|  |  |  |  |  |  |  |
|  |  |  |  |  |  |  |
|  |  |  |  |  |  |  |
| Family/species/ local name | Number of citations | | Use-value | | Part used | Disease (or illness) |
|  | JN | CR | JN | CR |  |  |
| Prochilodontidae  *Prochilodus nigricans* Agassiz, 1929 - black prochilodus, “curimatã” | 1 | 1 | 0.05 | 0.11 | Fat (5) | Cholesterol |
| Anostomidae  Leporinus steindachneri Eigenmann, 1907 - fish, piau | 1 | - | 0.05 | - | Fat (5) | Cholesterol |
| Syngnathidae  *Hippocampus reidi* (Ginsburg, 1933) – seahorse, “cavalo marinho” | 5 | - | 0.27 | - | Whole animal (1) | Asthma |
| Gymnotidae  *Electrophorus electricus* (Linnaeus, 1766) - electric eel, “peixe elétrico” | 1 | - | 0.05 | - | Fat (5) | Rheumatism and bruises |
| **Reptiles**  *Phrynops tuberosus* (Peters, 1870) - side-necked turtle, “cágado” | 12 | 6 | 0.66 | 0.66 | Fat (5) and shell (1) | Rheumatism, bruises (5) and asthma (1) |
| Alligatoridae  *Caiman crocodilus* (Linnaeus, 1758) - caiman, “jacaré-tinga” | - | 1 | - | 0.11 | Skin (1) | Asthma (1) |
| Viperidae  *Crotalus durissus* (Linnaeus, 1758) - rattle snake, “cascavel” | 13 | 5 | 0.72 | 0.55 | Fat (5) and rattle (6) | Rheumatism, osteoporosis, leprosy, back ache, fissures on feet, ear aches (5), “simpatias” (6) |
| Tropiduridae  *Tropidurus hispidus* Spix, 1825 - lava lizard, “lagartixa” | - | 2 | - | 0.22 | Whole animal (7) | Pityriasis |
| Teiidae  *Tupinambis merianae* (Duméril and Bibron, 1839) - teju lizard, “tiú” | 15 | 5 | 0.83 | 0.55 | Fat (5) | Rheumatism, inflammations, fissures on feet and ear aches |
| **Birds**  Cathartidae  *Coragyps atratus* (Bechstein, 1793) - black vulture, “urubu” | 7 | - | 0.38 | - | Liver (8) | Alcoholism |
| Phasianidae  *Gallus domesticus* Linnaeus, 1758 - chicken, “galinha” | 4 | 3 | 0.22 | 0.33 | Fat (5) | Sore throat |
| Family/species/ local name | Number of citations | | Use-value | | Part used | Disease (or illness) |
|  | JN | CR | JN | CR |  |  |
| *Pavo cristatus* Linnaeus, 1758 - pea-cock, “pavão” | - | 1 | - | 0.11 | Feather (1) | Asthma |
| Cuculidae  *Crotophaga ani* Linnaeus, 1758 - smooth-billed ani, “anu” | - | 1 | - | 0.11 | Whole animal (9) | Asthma |
| **Mammals**  Procyonidae  Procyon cancrivorus (Cuvier, 1798) crab-eating raccoon, “guaxinim” | 1 | - | 0.05 | - | Skin (7) | Snake bites |
| Canidae  *Cerdocyon thous* (Linnaeus, 1766)  fox, “raposa” | 4 | 1 | 0.22 | 0.11 | Fat (5) | Ear aches, inflammations and sore throat |
| Dasyponidae  *Euphractus sexcinctus* (Linnaeus, 1758), armadillo, “tatu peba” | 3 | 1 | 0.16 | 0.11 | Tail (10) and urine (2) | Ear aches, deafness (10) and urinary infections (2) |
| *Dasypus novemcinctus*, Linnaeus, 1758, nine-banded armadillo, “tatu galinha” | 2 | 1 | 0.11 | 0.11 | Tail (10) | Ear aches and deafness |
| Bovidae  *Ovis aries* (Linnaeus, 1758), sheep, “carneiro” | - | 2 | - | 0.22 | Fat (5) | Rheumatism, arthritis |
| *Bos taurus* (Linnaeus, 1758), domestic cattle, “boi” | 1 | 1 | 0.05 | 0.11 | Horn (1, 6), urine (8) and marrow (11) | Asthma (1), whooping cough (8), rheumatism (11) and “simpatias” (6) |
| Suidae  *Sus scrofa* (Linnaeus, 1758), pig, porco | - | 1 | - | 0.11 | Navel (1) | Bronchitis |
| Cervidae  *Mazama* sp. deer, “veado” | 4 | 1 | 0.22 | 0.11 | Hoof, horn (1) and tail (6) | “Simpatia” (6) and rheumatism (1) |

Legend: (1) Cook and macerate; (2) Prepare a tea with the animal part utilized and ingest it; (3) Ingest with other alternative medicines; (4) Similar to item three with the addition of wax; (5) Ingest directly or rub on the affected area; (6) Religious rituals or “simpatias”; (7) rub on the affected area; (8) Cook and macerate and ingest with some food; (9) Cook without salt and ingest; (10) Rub on the ear; (11) Ingest alone or mixed with some food; JN: Juazeiro do Norte; CR: Crato
